# Towards the phenotyping of autism spectrum disorder in children with tuberous sclerosis complex

**DOI:** 10.1111/dmcn.16328

**Published:** 2025-04-14

**Authors:** Ilaria Bonemazzi, Caterina Ancora, Veronica Ferasin, Marco Lunghi, Irene Toldo

**Affiliations:** ^1^ Department of Women's and Children's Health University of Padua – Pediatric Neurology and Neurophysiology Unit Padua Italy; ^2^ Azienda ULSS 8 Berica Veneto Italy

## Abstract

**This commentary is on the original article by Mitchell et al. on pages** 1165–1175 **of this issue.**

Autism is a common neurodevelopmental outcome in children with tuberous sclerosis complex (TSC), yet its phenotypic variability within this population remains poorly understood. Mitchell et al.^1^ focused on the in‐depth phenotyping of development in children with TSC, comparing those with autism to those without, which has high relevance to clinical practice and interventions. Although the authors worked with a small sample, they employed a comprehensive, multidimensional battery of tests and questionnaires aimed at exploring the most critical areas of development. The study covered a relatively broad time range (2016–2021), and it would have been interesting to apply the 2015 TSC‐associated neuropsychiatric disorders (TAND) checklist,^2^ specifically designed for assessing neuropsychiatric disorders associated with TSC.^2^, ^3^ Autism diagnosis was based on the Autism Diagnostic Observation Schedule, Second Edition (ADOS‐2) (which is the best standard) and DSM‐5 interview, associated with a multidisciplinary clinical judgment in unclear or discordant cases. This is a valid clinical approach, but cases with mild autism would be better assessed with other tools (i.e. Ritvo Autism Asperger Diagnostic Scale, Revised).^4^ There is also a risk of overestimating severe autism associated with severe intellectual disability.^4^

The main strength of the study^1^ was the multidimensional assessment of neurocognitive and adaptive functioning, based on standardized tests (ADOS‐2, Wechsler Preschool & Primary Scale of Intelligence/Wechsler Intelligence Scale for Children, Preschool Language Scales, Peabody Picture Vocabulary Test, Clinical Evaluation of Language Fundamentals) and on semi‐structured questionnaires for parents (Vineland Adaptive Behavior Scale, Behavior Rating Inventory of Executive Function, Conners, Child Behavior Checklist). In the future, it would be useful to enhance this protocol with a comprehensive neuropsychological evaluation using specific tests (e.g. NEPSY‐II),^5^ since attention and executive functions cannot be accurately quantified only through parent reports.

The findings are informative and useful, although it is unclear whether the diagnosis of autism in the study population was made before or during the study. The age range is quite broad (2 years 6 months–16 years), and the age at autism diagnosis is not reported. It is also noteworthy that the sample is unexpectedly skewed toward females. The authors highlight the importance of assessing language disorders and attention‐deficit/hyperactivity disorder symptoms in this context. These should be thoroughly investigated using diagnostic instruments rather than screening tools (e.g. use of the Conners 3–Parent Short Form would be insufficient).

It is surprising that Mitchell et al. did not find a marked difference in adaptive functioning between autistic and non‐autistic children, both with and without intellectual disability. This could be due to the small sample size (e.g. 6 vs 13 without intellectual disability), calling for analyses of larger samples stratified by age and cognitive functioning.

This important study points to research needs to be met in future studies on development in children with TSC, particularly the use of a variety of diagnostic tools in addition to medical history and direct clinical observation, and documentation of developmental trajectories from the time of diagnosis through longitudinal evaluations. Implementing standardized, multidisciplinary clinical protocols would allow for data comparison across large cohorts of children with TSC.

## FUNDING INFORMATION

No funding for this article.

## CONFLICT OF INTEREST STATEMENT

We declare no conflicts of interest.

## Data Availability

Not required.
